# A randomized controlled clinical trial of vildagliptin plus metformin combination therapy in patients with type II diabetes mellitus

**DOI:** 10.3892/etm.2014.1545

**Published:** 2014-02-13

**Authors:** YONG SU, YA-LI SU, LI-FANG LV, LI-MIN WANG, QUAN-ZHONG LI, ZHI-GANG ZHAO

**Affiliations:** Department of Endocrinology, Henan Provincial People’s Hospital, Zhengzhou University, Zhengzhou, Henan 450003, P.R. China

**Keywords:** type II diabetes mellitus, vildagliptin, metformin, body weight, blood lipid, hepatorenal function

## Abstract

The aim of the present study was to assess the efficacy and safety of vildagliptin plus metformin combination therapy in patients with type II diabetes mellitus. Type II diabetic patients with poor glycemic control following at least three months of metformin treatment were selected and randomized into two groups. Vildagliptin or placebo was administered with metformin. Body weight, fasting blood glucose (FBG), postprandial glucose (PPG), glycated hemoglobin (HbA1c), blood lipid and hepatorenal function levels were analyzed in the patients prior to and 24-weeks after the trial. FBG, PPG and HbA1c levels of the patients in the vildagliptin group significantly decreased following the trial, whereas no statistically significant differences were observed in the various indicators of the placebo group prior to and following the trial. The FBG, PPG and HbA1c levels in the vildagliptin group were significantly lower compared with the placebo group 24-weeks after the trial. Comparisons of body weight, blood lipid and hepatorenal function between the groups prior to and following the trial exhibited no statistically significant differences. Therefore, vildagliptin plus metformin combination therapy effectively reduced FBG, PPG and HbA1c levels in patients with no risk of weight gain or hepatorenal dysfunction.

## Introduction

The incidence of type II diabetes mellitus is gradually increasing. According to the International Diabetes Federation (1), the number of diabetic patients worldwide is likely to reach 552 million in 2030, in which 90% of these patients have type II diabetes.

Vildagliptin is a highly selective, reversible and competitive dipeptidyl peptidase-4 (DPP-4) inhibitor. The efficacy of vildagliptin plus metformin combination therapy has been demonstrated to lower the risk of weight gain and hypoglycemia and significantly decrease fasting blood glucose (FBG), postprandial glucose (PPG) and glycated hemoglobin (HbA1c) levels. No evidence has shown that vildagliptin use carries the risk of hepatorenal dysfunction (2–7). In 2009, the American Society for Clinical Endocrinology released the diabetes control guidelines (8,9), which indicate DPP-4 inhibitors as first-line drugs for type II diabetes. In addition, according to the guidelines, if metformin or thiazolidinediones do not produce favorable results, a DPP-4 inhibitor may be used in combination to support the therapy.

However, no associated studies exist regarding this combination therapy in type II diabetes patients in China. Thus, in the present study, the effects of vildagliptin plus metformin combination therapy in decreasing the blood glucose level were analyzed. In addition, the effects of this therapy on body weight and hepatorenal function of patients were analyzed.

## Materials and methods

### Subjects

A total of 600 patients (males, 324; females, 276) were involved in the trial. The study was conducted in accordance with the Declaration of Helsinki and with approval from the Ethics Committee of Henan Provincial People’s Hospital (Zhengzhou, China). Written informed consent was provided by all participants.

### Inclusion criteria

Patients diagnosed with type II diabetes with a 75-g oral glucose tolerance test that were aged between 18 and 70 years-old were included in the study. In addition, the included patients had been treated with metformin and A-glycosidase inhibitors for longer than three months, had HbA1c levels >6.5%, FBG levels >7.0 mmol/l and normal hepatorenal function. All subjects volunteered to participate in this study and provided informed consent.

### Exclusion criteria

Patients excluded from the study had type I or other specific forms of diabetes, were younger than 18 or older than 70 years-old or had used hypoglycemic agents other than metformin and A-glycosidase inhibitors. Patients with diabetic ketoacidosis, hyperglycemic nonketotic hyperosmolar syndrome, chronic complications that required insulin treatment or patients that required insulin treatment under stress were also excluded. Patients with evident liver or kidney disease [alanine aminotransferase (ALT) or aspartate aminotransferase (AST) levels >3 times the normal upper limit, total bilirubin levels >1.5 times the normal upper limit or creatinine (CREA) levels >115 μmol/l], pregnant or lactating women and patients with poor compliance were also excluded from the study.

### Diet design

Participants received individual diet guidance from an endocrinologist. Total daily calorie intake from food, including 50% carbohydrate, 15% protein and 35% fat, for each subject was calculated according to 30 kcal/kg of ideal weight [ideal weight (kg) = measured weight (kg) − 105]. Each subject was assigned to three meals with calorie intake assigned as follows: 1/5, 2/5 and 2/5. Mealtimes and meal content were relatively uniform, and intake of alcohol, strong tea or coffee was prohibited during the trial.

### Drug treatment

Subjects randomly received vildagliptin (100 mg/day orally in two equal doses) or placebo and received drugs according to prescription. Patients who forgot or adjusted drug doses or treatment were excluded from the trial. Patients self-monitored and recorded fingerstick blood glucose and hypoglycemic events. The body weight, FBG, PPG, HbA1c, blood lipid and hepatorenal function levels of the patients 24-weeks after the trial were analyzed. Drugs were supplied by Henan Pharmaceutical Co., Ltd. (Zhengzhou, China).

### Statistical analysis

Data are presented as mean ± SD. The data of the groups obtained before and after the trial were compared using a Student’s t-test. A paired t-test was used to compare associated indicators prior to and following the trial. Data were analyzed with SPSS 13.0 software (SPSS, Inc., Chicago, IL, USA) and P<0.05 was considered to indicate a statistically significant difference.

## Results

### Clinical characteristics

A total of 600 patients (males, 324; females, 276), including 300 in the vildagliptin group and 300 in the placebo group, were included in the study. The patients had an average age of 48.14±12.986 years (18–70 years), body weight of 64.40±10.057 kg (45–80 kg), FBG level of 9.04±1.568 mmol/l (7.0–12.0 mmol/l), mean postprandial glucose (MPPG) level of 10.28±1.568 mmol/l (7.0–13.0 mmol/l) and HbA1c level of 8.59±1.543% (6.5–11.6%). In total, 14 patients quit the trial: Four violated the treatment and two were lost to follow-up in the vildagliptin group, while five violated the treatment, two were lost to follow-up and one had an adverse event in the placebo group. A total of 294 and 292 patients completed the clinical trial in the vildagliptin and placebo groups, respectively.

Table I shows that there were no statistically significant differences in age, body weight, FBG, MPPG, HbA1c, blood lipid or hepatorenal function between the two groups (P>0.05).

### Comparison of associated indicators

The patients underwent examinations 24-weeks after the trial. In the vildagliptin group, the FBG, MPPG and HbA1c levels significantly decreased (P<0.05), whereas the body weight, blood lipid and hepatorenal function did not significantly change following the trial (P>0.05). No statistically significant differences were observed in the various indicators in the placebo group before and after the trial (Table II).

### Comparison of efficacy

Table III shows that 24-weeks after the trial, the FBG, PPG and HbA1c levels in the vildagliptin group were significantly lower compared with the placebo group (P<0.05). Comparisons of body weight, blood lipid and hepatorenal function between the groups exhibited no statistically significant differences.

## Discussion

Vildagliptin is a highly selective and reversible substrate-like DPP-4 inhibitor that binds to and forms a complex with DPP-4 to inhibit DPP-4 activity, increase active glucagon peptide-1 (GLP-1) concentration, promote glucose-dependent insulin secretion from pancreatic β cells and simultaneously adjust the function of α cells to reduce glucagon levels during hyperglycemia (8) to achieve bidirectional regulation of blood sugar. Vildagliptin plus metformin combination therapy improves glycemic control in type II diabetic patients with favorable safety and tolerance levels (2,5). Metformin may increase GLP-1 synthesis, whereas vildagliptin inhibits DPP-4 and increases activated GLP-1 levels. Therefore, the synergistic effect of vildagliptin and metformin maximizes activated GLP-1 levels and further increases insulin secretion and sensitivity (10).

The efficacy of vildagliptin plus metformin combination therapy has been reported in numerous studies in various countries (4,5,11–18). However, this therapy has not been reported in China. Thus, the aim of the present clinical trial was to observe any decrease in blood glucose level caused by this combined therapy, as well as any adverse effects of vildagliptin. During the study, two cases of hypoglycemia and six incomplete cases in the vildagliptin group were observed. By contrast, in the placebo group, no hypoglycemia case was observed and eight patients quit.

The present study revealed that vildagliptin treatment markedly decreased FBG, PPG and HbA1c levels in patients when compared with patients prior to vildagliptin treatment or following placebo treatment (Tables II and III). In addition, no significant change in body weight was observed. The results indicate that the total cholesterol (CHOL), triglyceride (TG), high density lipoprotein (HDL) and low density lipoprotein (LDL) levels of patients decreased and the ALT, AST, UREA and creatine (CREA) levels increased following vildagliptin treatment.

Bidirectional regulation of vildagliptin not only reduces FBG levels, but also reduces PPG to reduce HbA1c levels effectively. Retrospective studies have demonstrated that DPP-4 inhibitor levels correlate with decreased CHOL (19). No correlation was observed between vildagliptin and increased risk of adverse liver conditions or elevated hepatase (3,7,20,21). The present study demonstrated that CHOL, TG, HDL and LDL levels decreased and hepatase levels slightly increased following vildagliptin treatment, which may be attributed to the small sample size or the effect of a combination of hypoglycemic agents. However, no statistically significant differences were observed in these indicators before and after vildagliptin treatment or after placebo treatment. These results indicate that an increase in ALT due to vildagliptin intake does not negatively affect the liver function of patients. In addition, both UREA and CREA levels increased following vildagliptin treatment but were not statistically different from those prior to vildagliptin treatment. This result indicates that vildagliptin does not negatively affect the kidney function of patients.

In conclusion, vildagliptin plus metformin combination therapy effectively reduces the FBG, PPG and HbA1c levels and increases the hepatase and CREA levels of patients with type II diabetes. The combination therapy does not negatively affect hepatorenal functions, but reduces blood lipid level with a low risk of weight gain and hypoglycemia. Independent of safety or efficacy, vildagliptin may be considered as a useful hypoglycemic drug for type II diabetic patients with poor glycemic control following metformin monotreatment.

## Figures and Tables

**Table I:** Clinical characteristics of the subjects.

Characteristics	Vildagliptin group (mean ± SD)	Placebo group (mean ± SD)	t-value	P-value
Age, years	47.61±14.409	48.67±11.564	−0.215	0.813
Height, cm	165.50±6.456	164.61±6.456	0.778	0.486
Weight, kg	65.14±10.317	63.67±9.794	0.335	0.656
FBG, mmol/l	9.22±1.691	8.86±1.445	0.864	0.389
MPPG, mmol/l	10.23±1.657	10.33±1.395	0.038	0.883
HbA1c, %	8.76±1.718	8.42±1.368	0.343	0.671
ALT, U/l	30.44±15.846	23.38±9.928	1.626	0.113
AST, U/l	22.89±7.676	20.67±5.246	1.017	0.327
CHOL, mmol/l	4.96±1.009	4.53±1.096	1.001	0.323
TG, mmol/l	1.93±1.383	1.85±1.373	0.176	0.834
HDL, mmol/l	1.18±0.331	1.08±0.287	1.420	0.178
LDL, mmol/l	2.77±0.671	2.45±0.836	1.308	0.213
UREA, mmol/l	5.58±1.652	5.67±1.366	−0.181	0.749
CREA, μmol/l	57.18±13.367	60.57±20.359	−0.590	0.559

SD, standard deviation; FBG, fasting blood glucose; MPPG, mean postprandial glucose; HbA1c, glycated hemoglobin; ALT, alanine aminotransferase; AST, aspartate aminotransferase; CHOL, total cholesterol; TG, total triglycerides; HDL, high density lipoproteins; LDL, low density lipoproteins; UREA, urea; CREA, creatinine.

**Table II:** Comparison of associated indicators before and after vildagliptin or placebo treatments.

Indicator	Before treatment (mean ± SD)	After treatment (mean ± SD)	t-value	P-value
Vildagliptin group (n=294)				
Weight, kg	65.14±10.317	65.84±10.454	0.066	0.935
FBG, mmol/l	9.22±1.691	6.52±0.702	6.288	<0.0001
MPPG, mmol/l	10.23±1.657	8.13±0.635	6.040	<0.0001
HbA1c, %	8.76±1.718	6.65±1.251	4.261	<0.0001
ALT, U/l	30.44±15.846	33.94±11.275	2.343	0.129
AST, U/l	22.89±7.676	27.23±6.857	1.245	0.251
CHOL, mmol/l	4.96±1.009	4.50±0.882	1.517	0.138
TG, mmol/l	1.93±1.383	1.36±0.853	1.539	0.133
HDL, mmol/l	1.18±0.331	1.15±0.239	0.385	0.713
LDL, mmol/l	2.77±0.671	2.58±0.731	1.267	0.214
UREA, mmol/l	5.58±1.652	5.91±2.089	−0.370	0.724
CREA, μmol/l	57.18±13.367	57.21±14.564	0.036	0.972
Placebo group (n=292)				
Weight, kg	63.67±9.794	64.69±9.381	−0.009	0.993
FBG, mmol/l	8.86±1.445	8.60±1.274	0.573	0.572
MPPG, mmol/l	10.33±1.395	9.70±1.348	1.539	0.136
HbA1c, %	8.42±1.368	8.26±1.180	1.061	0.296
ALT, U/l	23.38±9.928	26.89±8.065	−1.198	0.249
AST, U/l	20.67±5.246	23.67±4.666	−1.867	0.071
CHOL, mmol/l	4.53±1.096	4.77±0.950	−0.132	0.904
TG, mmol/l	1.85±1.373	1.99±1.327	−0.099	0.932
HDL, mmol/l	1.08±0.287	1.24±0.501	−1.992	0.054
LDL, mmol/l	2.45±0.836	2.67±0.812	−0.468	0.657
UREA, mmol/l	5.67±1.366	5.56±1.419	0.922	0.363
CREA, μmol/l	60.57±20.359	64.61±21.947	−0.559	0.580

SD, standard deviation; FBG, fasting blood glucose; MPPG, mean postprandial glucose; HbA1c, glycated hemoglobin; ALT, alanine aminotransferase; AST, aspartate aminotransferase; CHOL, total cholesterol; TG, total triglycerides; HDL, high density lipoproteins; LDL, low density lipoproteins; UREA, urea; CREA, creatinine.

**Table III:** Comparison of indicators between vildagliptin and placebo groups 24-weeks after the trial (mean ± SD).

Indicator	Vildagliptin group (n=294)	Placebo group (n=292)	t-value	P-value
Weight, kg	65.84±10.454	64.69±9.381	0.367	0.709
FBG, mmol/l	6.52±0.702	8.60±1.274	−6.125	<0.0001
MPPG, mmol/l	8.13±0.635	9.70±1.348	−4.785	<0.0001
HbA1c, %	6.65±1.251	8.26±1.180	−3.743	0.001
ALT, U/l	33.94±11.275	26.89±8.065	2.351	0.427
AST, U/l	27.23±6.857	23.67±4.666	2.238	0.123
CHOL, mmol/l	4.50±0.882	4.77±0.950	−0.544	0.563
TG, mmol/l	1.36±0.853	1.99±1.327	−1.718	0.097
HDL, mmol/l	1.15±0.239	1.24±0.501	−0.987	0.342
LDL, mmol/l	2.58±0.731	2.67±0.812	−0.360	0.732
UREA, mmol/l	5.91±2.089	5.56±1.419	0.937	0.358
CREA, μmol/l	57.21±14.564	64.61±21.947	−1.308	0.235

SD, standard deviation; FBG, fasting blood glucose; MPPG, mean postprandial glucose; HbA1c, glycated hemoglobin; ALT, alanine aminotransferase; AST, aspartate aminotransferase; CHOL, total cholesterol; TG, total triglycerides; HDL, high density lipoproteins; LDL, low density lipoproteins; UREA, urea; CREA, creatinine.
